# Bio-Organic Fertilizer with *Bacillus velezensis* Promoted Plant Growth by Regulating Soil Microbial Community Structure and C/N Cycle Function

**DOI:** 10.3390/plants15030382

**Published:** 2026-01-26

**Authors:** Haiyun Zhang, Cuixue Cui, Shuangxi Li, Weiguang Lv, Juanqin Zhang, Xianpu Zhu, Chenglong Xu, Qun Wang, Naling Bai, Hanlin Zhang

**Affiliations:** 1 Institute of Eco-Environmental Protection, Shanghai Academy of Agricultural Sciences, Shanghai 201403, China; zhanghaiyun@saas.sh.cn (H.Z.);; 2 Shanghai Agri-Environmental and Cultivated Land Conservation of Scientific Observation and Experiment Station, Ministry of Agriculture and Rural Affairs, Shanghai 201403, China; 3 Key Laboratory of Low-Carbon Green Agriculture in Southeastern China, Ministry of Agriculture and Rural Affairs, Shanghai 201403, China; 4 School of Chemical and Environmental Engineering, Shanghai Institute of Technology, Shanghai 201418, China; 5 Key Laboratory of Recycling and Eco-Treatment of Waste Biomass of Zhejiang Province, School of Environmental and Natural Resources, Zhejiang University of Science and Technology, Hangzhou 310023, China

**Keywords:** bio-organic fertilizer, microorganism, carbon cycle, nitrogen cycle, functional gene

## Abstract

This study investigated the effects of bio-organic fertilizer (BF) containing plant growth-promoting bacterium *Bacillus velezensis* SS-20 on soil properties, microbial community structure, and C/N cycle functional genes. The results showed that compared with chemical fertilizer (CF) and deactivated bio-organic fertilizer (BFD) treatments, BF significantly improved soil physicochemical properties. Soil pH, organic matter, total nitrogen, total potassium, and total phosphorus content under BF treatment were increased by 14.8%, 56.5%, 48.2%, 38.8%, and 58.4%, respectively, compared to the control; soil urease and sucrase activities increased by 3.5 and 2.4 times those of CF treatment, respectively. Meanwhile, BF increased pakchoi yield by 11.2% (vs. CF). BF treatment enhanced the relative abundance of beneficial bacteria *Actinomycetota* by 28.4% compared with the BFD treatment and raised that of fungi *Ascomycota* to 1.9 times that of the control. At the genus level, BF significantly enriched biocontrol-relevant genus *Pseudogymnoascus*, whose abundance reached three times that of CF treatment, while the abundance of potentially harmful genus *Penicillium* decreased by 82%. BF also led to a high degree of synergy in carbon and nitrogen cycles. Functional gene analysis indicated that BF down-regulated multiple carbon-degrading genes, increased organic nitrogen metabolism genes by 5.3%, and reduced denitrification genes by 13.3%. Overall, bio-organic fertilizer optimized the soil microenvironment, regulated the microbial community structure, and improved C/N use efficiency and plant growth by introducing functional microorganisms and organic matter.

## 1. Introduction

The excessive application of chemical fertilizer leads to soil acidification, reduced microbial diversity, and an imbalance in the carbon and nitrogen cycle, which poses a serious threat to soil health and agricultural sustainability [1,2]. Bio-organic fertilizers, rich in organic matter and functional microorganisms, are widely studied for their capability of improving soil nutrient cycling and promoting plant growth through core functional bacteria such as nitrogen fixers (*Rhizobium*, *Azotobacter*, *Nostoc*), phosphate solubilizers (*Bacillus megaterium*, *Pseudomonas fluorescens*), potassium solubilizers (*B. mucilaginosus*), biocontrol agents (*Trichoderma harzianum*, *Streptomyces*), and plant growth promoters (*Glomus*, *Bacillus amyloliquefaciens*).

The regulatory mechanisms of bio-organic fertilizer on plant growth promotion are multidimensional, including positive physiological responses of plants, particularly under biotic and abiotic stress, improved soil nutrition availability, and optimized soil microbial community and functions [3,4,5,6,7,8]. Some research documented that bio-organic fertilizers improved soil nutrition and reshaped microbial communities. For instance, the long-term application of bio-organic fertilizer can significantly increase soil labile organic carbon components and enhance carbon utilization efficiency by regulating the microbial community structure [9,10]. Our previous study found that *Bacillus subtilis* A-5 significantly altered the rhizospheric bacterial community structure (especially *Bacillus*, *Micrococcaceae,* and *Nocardioides*) and enhanced the yield of Chinese cabbage [11]. Co-inoculation of protist and *Bacillus* promoted plant growth by reshaping rhizosphere bacterial community composition, with *Cellvibrio* as the most important predictor of plant growth [12]. Bio-organic fertilizers also play a significant role in optimizing soil microbial functions of the carbon and nitrogen cycle [13,14]. For instance, organic cultivation increased soil pH and available nutrient contents, alleviated microbial carbon and nitrogen limitations, and consequently improved microbial stoichiometric homeostasis and carbon use efficiency [15]. Functional microorganisms in bio-organic fertilizers, such as *Bacillus subtilis*, can significantly reduce ammonia volatilization loss by enhancing soil ammonia oxidation (increased functional genes and bacteria) and inhibiting urea hydrolysis (reduced *ureC* gene), while simultaneously improving nitrogen uptake efficiency. This highlights the key role of functional bacteria in regulating nitrogen transformation and mitigating greenhouse gas emissions [16]. *Bacillus subtilis* A-5 and its fermentation broth were found to impact relative abundances of important bacterial taxa related to metabolite degradation, nitrogen cycling, and predation, thereby significantly facilitating plant growth [11]. A consortium of *B*. *velezensis* SQR9 and protists has been proven to enhance plant growth by enhancing bacterial functional genes of nitrogen metabolism [12].

Overall, the mechanistic study of how bio-organic fertilizers promote plant growth mainly addressed their effects on soil properties and microbial communities, with several studies investigating the effects on carbon or nitrogen transformation. However, bio-organic fertilizers’ effects on the soil carbon and nitrogen cycle, especially C-N cycle interactions at a genetic level, remain insufficiently clear, which is important for interpreting plant nutrition availability and growth promotion. In this study, a field plot experiment was conducted to investigate the effects of bio-organic fertilizer (containing the functional bacterium *Bacillus velezensis* SS-20) on the growth of Chinese cabbage, soil microbial community structure, and C/N cycle functions. The turnover of carbon and nitrogen can be reflected by the functional genes of soil microbial communities. Therefore, an abundance of genes related to the carbon cycle (including carbon fixation, methane metabolism, and carbon degradation) and nitrogen cycle (including nitrogen fixation, nitrification, denitrification, and ammonification) were determined [17,18,19,20,21,22]. It is hypothesized that bio-organic fertilizer with *Bacillus velezensis* SS-20 can facilitate plant growth not only through improving soil nutrition and reshaping soil microbes but also by impacting the C/N cycle and C-N interactions. The objective is to reveal the responses and underlying linkages among plant growth, soil properties, microbial communities, and the C/N cycle, thereby providing theoretical support for optimizing fertilization management strategies, enhancing soil fertility, and strengthening soil carbon sequestration capacity.

## 2. Materials and Methods

### 2.1. Test Materials

The chemical fertilizer (N:P:K = 15:15:15) was purchased from Shanghai Zhongde Fertilizer Co., Ltd. (Shanghai, China). The bio-organic fertilizer was produced by Shanghai Ouhai Energy Technology Co., Ltd. (Shanghai, China). and fermented by *Bacillus velezensis* SS-20, a strain with stress resistance and growth promotion function. The organic matter content of bio-organic fertilizer is 53.25%, the total nutrient (N + P_2_O_5_ + K_2_O) content is 6.8%, the viable bacteria are ≥200 million/g, and the pH is 7.6. The deactivation method of bio-organic fertilizer is as follows: the bio-organic fertilizer was spread with a thickness of about 3–5 cm when the sunlight was sufficient and exposed for two days. When the sunlight was insufficient, a UV lamp was used to supplement irradiation and the fertilizer was turned over every 2 h. The tested plant is *Brassica chinensis* L., and the specific variety is Shanghaiqing.

### 2.2. Experimental Design

The experiment was conducted in the greenhouse of Shanghai Huiqun Vegetable Planting Professional Cooperative (121°33′40′′ E, 30°56′48′′ N) in the Fengxian District of Shanghai from 30 September to 1 December 2024. Before the experiment, (on 21 September 2024), five soil samples of 0–20 cm depth from each plot were collected, and the samples were fully homogenized into a single one (approximately 2 kg), which was air-dried for analyzing soil background physicochemical properties. Soil properties are summarized in Table 1 as follows, with pH being 6.16, organic matter content being 11.19 g/kg, total nitrogen content being 1.31 g/kg, total phosphorus content being 1.14 g/kg, and total potassium content being 1.52 g/kg.

CF (chemical fertilizer), BF (bio-organic fertilizer), and BFD (deactivated bio-organic fertilizer) treatments, as well as the control (CK), were set in the experiment. Each treatment contained three replicates. The experimental plot for each treatment was 3 m × 15 m, and all treatments were arranged in random blocks. The base fertilizer for CF treatment was 750 kg/ha of CF and was 300 kg/ha of CF + BFD (60% chemical nitrogen replacement) for BFD treatment. That for BF treatment was 300 kg/ha of CF + BFD (60% chemical nitrogen replacement). Each fertilization treatment maintained the same amount of base nitrogen. All treatments used urea topdressing three times during the growth period, and the application amount was the same. The tested plant was sown on the first day after fertilization, thinned out with a space of 8–10 cm, and grown for 60 days. The plant yield was measured by weighing the actual biomass of pakchoi from each experimental plot after harvest. Soil samples were collected from each plot using the same method (as described above) on 1 December 2024. One portion was air-dried for physicochemical property analysis, and the other portion was stored at −80 °C for enzyme activity and metagenomic analysis.

### 2.3. Sample Analysis

#### 2.3.1. Analysis of Soil Properties and Enzyme Activities

Soil pH was determined by the potentiometric method (soil-to-water ratio of 1:2.5). Soil organic matter was determined by the potassium dichromate oxidation method (treated with excessive K_2_Cr_2_O_7_-H_2_SO_4_ solution, and the remaining K_2_Cr_2_O_7_ was titrated with FeSO_4_ standard solution). Soil total nitrogen (TN) was analyzed by the Kjeldahl method [23], which is completely converted into ammonium nitrogen by an oxidation–reduction reaction, and then the ammonia distilled by alkalization is absorbed by boric acid (titrated with standard HCl solution). Soil total phosphorus (TP) was digested by HNO_3_, HClO_4,_ and HF to orthophosphate and then analyzed by ICP-AES. Soil total potassium (TK) was also digested by HNO_3_, HClO_4,_ and HF and then analyzed by ICP-AES. Soil enzyme activities were determined using kits purchased from Suzhou Keming Biotech Inc. (Suzhou, China). Specifically, soil urease activity was analyzed by the indophenol blue colorimetry method, quantifying the NH_3_-N amount produced by urease hydrolysis of urea. Under alkaline conditions, NH_3_-N reacts with phenol and hypochlorite to form blue indophenol, which can be measured with a spectrophotometer at a wavelength of 578 nm. Soil catalase activity was determined by measuring absorption attenuation (at 240 nm wavelength) of H_2_O_2_ catalyzed by soil catalase. Soil sucrase activity was determined by the 3,5-Dinitrosalicylic acid method. Soil sucrase catalyzes the degradation of sucrose to produce reduced sugar, which further reacts with 3,5-dinitrosalicylic acid to produce a brownish red amino compound with characteristic light absorption at 510 nm.

#### 2.3.2. Soil DNA Extraction and Metagenome Sequencing

Soil DNA was extracted by the Fastpure soil DNA isolation kit (magnetic bead) (MJYH, Shanghai, China). DNA concentration and purity were detected, and the integrity was tested by 1% agarose gel electrophoresis. The qualified DNA was broken into 400 bp fragments by ultrasonic crusher (Gene Co., Ltd., Shanghai, China), and the PE library was constructed by the NEXTFLEXTM Rapid DNA-Seq Kit (Bioo Scientific, Austin, TX, USA). Finally, it was amplified by bridge PCR, denatured, hybridized, and extended, and then amplified linearly. The metagenome was sequenced by the NovaSeq X Plus (Illumina Inc., San Diego, CA, USA) sequencing platform (Shanghai Majorbio Bio-pharm Technology Co., Ltd., Shanghai, China).

### 2.4. Data Analysis

SPSS 26.0 one-way analysis of variance and the LSD multiple comparison method were used to test the effects of various fertilizers on soil physicochemical properties, enzyme activities, and C/N cycle functional genes. Bioinformatic analysis was performed using the Majorbio Cloud (www.majorbio.com). PCoA analysis was conducted for soil bacterial and fungal communities, along with Permanova, Anosim, and pair test for each treatment. The difference in microbial community composition among various treatments was tested using one-way anova and Tukey’s test. According to the marked gene set, the C/N cycle genes of each sample were selected from KEGG functional annotation, and the functional composition was analyzed based on RPKM abundance. RDA analysis was conducted between C/N cycle gene abundance and soil physicochemical properties/enzyme activities. LEfSe analysis of soil bacterial and fungal communities under various fertilizer treatments was performed to find out the distinct taxa among groups (LDA > 4.0, *p* < 0.05).

## 3. Results

### 3.1. Effect of Bio-Organic Fertilizer on Soil Properties and Plant Growth

As shown in Table 2, soil pH was in the range of 4.99–5.85, which is weakly acidic. The soil pH of BF and BFD treatment was significantly higher than that of CK and CF. This indicates that chemical fertilizer aggravated soil acidification, while bio-organic fertilizer significantly alleviated acidification, which may be related to its rich organic acid anions, exchangeable bases, and humus buffer system. The soil organic matter and nutrients increased with applications of BF and BFD. Compared with CK, the contents of soil organic matter, total nitrogen, total potassium, and total phosphorus in BF treatment increased by 56.5%, 48.2%, 38.3%, and 58.4%, respectively. The contents of soil organic matter, total nitrogen, and total phosphorus in BFD treatment increased by 39.5%, 40.2%, and 46.8%, respectively, and total potassium showed an increasing trend but was not significantly different from the control. The contents of soil organic matter and total nitrogen in BF treatment were significantly higher than those from BFD treatment, while total phosphorus and total potassium were comparable with CK.

The application of bio-organic fertilizer significantly enhanced the activities of soil catalase, urease, and sucrase (Table 3), while CF treatment significantly reduced their activities, following the trend of BF > BFD > CK > CF. The activities of soil enzymes in BF treatment were significantly higher than those from other treatments (*p* < 0.05). Soil catalase activity was 1.2, 1.8, and 1.5 times that of BFD, CF, and CK, respectively. Soil urease activity was 1.3, 3.5, and 2.9 times that of BFD, CF, and CK, respectively. Soil sucrase activity was 1.4, 2.4, and 1.6 times that of BFD, CF, and CK, respectively.

All the fertilizers had significant effects on pakchoi yield (Table 2). The pakchoi yield treated with BF was the highest (28,047.10 kg/ha), which is significantly higher than that of the control and the other two treatments. This indicates that BF had the best efficiency, but there was no significant difference between BFD and CF treatment. Additionally, pakchoi yield was significantly positively correlated with soil nutrients (organic matter, total nitrogen, total phosphorus, total potassium) and urease activity (*p* < 0.05, Table S1).

### 3.2. Effects of Bio-Organic Fertilizer on Soil Bacterial and Fungal Diversity

As shown in Table S2, the *α* diversity of soil bacteria and fungi in each treatment was not significantly different from that of the control. Only the Chao index of soil fungi in BFD increased by 13.0% and 16.8% compared with CK and CF treatment, respectively.

**Figure 1:**
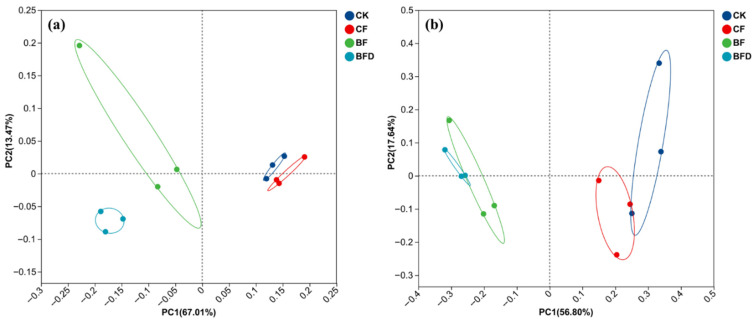
PCoA analysis of soil bacteria (**a**) and fungi (**b**) under various fertilizer treatments. CK: soil without any fertilizer; CF: chemical fertilizer; BF: bio-organic fertilizer; BFD: deactivated bio-organic fertilizer.

The structure of the soil bacterial community from various fertilizer treatments was significantly different, as shown in the PCoA results (Figure 1a). The first and second coordinate axes explained 67.01% and 13.47% of the overall change, respectively. BF, BFD, CK, and CF were significantly separated along the first coordinate axis (PC1). The soil fungal community was also significantly altered by various fertilizer treatments (Figure 1b). The first and second coordinate axes explained 56.8% and 17.64% of the overall change, respectively. BF, BFD, CK, and CF were significantly separated along the first coordinate axis (PC1).

Overall, BF and BFD treatments were distinct from CK and CF treatments for soil microbial communities. The Permanova test showed significant differences in bacterial (*p* = 0.001) and fungal (*p* = 0.005) communities among various groups. Anosim analysis also showed distinct bacterial (*r* = 0.74, *p* = 0.001) and fungal (*r* = 0.69, *p* = 0.001) communities among various groups. Beta diversity difference analysis (Student’s *t*-test) of bacteria and fungi was also conducted between various groups based on Bray–Curtis distance. Significant differences (*p* < 0.05) were found between CF and BFD treatment, and also between BF and BFD treatment for the bacteria community, and between BFD and CK for the fungal community.

### 3.3. Effects of Bio-Organic Fertilizer on Bacterial Community Composition

**Figure 2:**
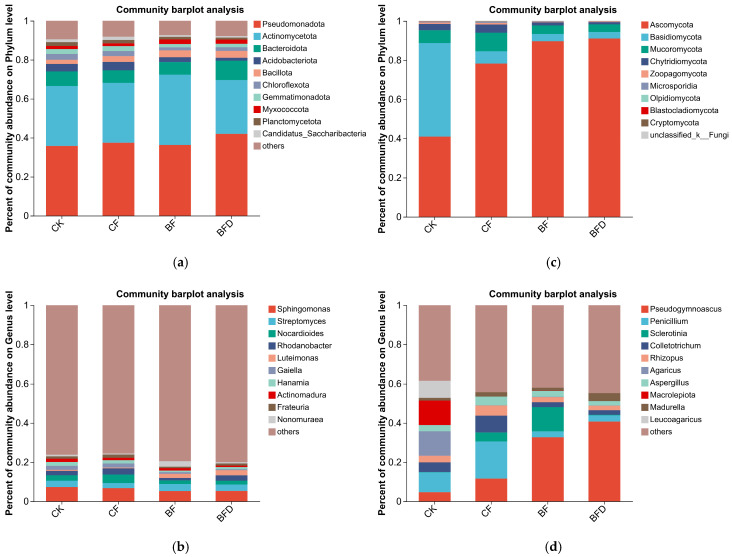
Community composition of soil bacteria at the phylum (**a**) and genus (**b**) levels and of soil fungi at the phylum (**c**) and genus (**d**) levels under various fertilizer treatments. CK: soil without any fertilizer; CF: chemical fertilizer; BF: bio-organic fertilizer; BFD: deactivated bio-organic fertilizer.

*Pseudomonadota*, *Actinomycetota,* and *Bacteroidota* were the dominant phyla in all treatments, and their relative abundances were 74.6–79.3% (Figure 2a). Compared with the control, BFD treatment increased *Bacteroidota* abundance by 47.9% (*p* < 0.01), while decreasing *Chloroflexota* and *Gemmatimonadota* abundance by 34.2% (*p* < 0.05) and 25% (*p* < 0.05), respectively. Under BF treatment, the relative abundance of *Chloroflexota* and *Gemmatimonadota* decreased by 40.7% (*p* < 0.05) and 26.1% (*p* < 0.05), respectively. Compared with CF treatment, BFD treatment increased the relative abundance of *Bacteroidota* by 67.1% (*p* < 0.01), and decreased the abundance of *Acidobacteriota* and *Gemmatimonadota* by 65.7% (*p* < 0.05) and 27.7% (*p* < 0.05), respectively. BF treatment reduced *Gemmatimonadota* abundance by 28.7% (*p* < 0.05). Compared with BFD treatment, BF reduced *Bacteroidea* abundance by 31.6% (*p* < 0.01), and increased that of *Actinomycetota* by 28.4% (*p* < 0.05), respectively.

As shown in Figure 2b, the composition of soil bacteria in each treatment was highly dispersed at the genus level. The relative abundance of the top-ten genera accounted for only about 20%, among which *Sphingomonas* was the most dominant, followed by *Streptomyces* and *Nocardioides*. Compared with the control, BFD treatment increased the relative abundance of *Luteimonas* by 313.7% and decreased *Gaiella* and *Actinomadura* by 74.2% (*p* < 0.05) and 57.2% (*p* < 0.05), respectively. *Luteimonas* in BF treatment increased by 212.8% (*p* < 0.001). Compared with CF, the relative abundance of *Nocardioides* and *Gaiella* in BFD treatment decreased by 53.6% (*p* < 0.05) and 75% (*p* < 0.05), respectively, and *Luteimonas* abundance reached six times that of CF treatment (*p* < 0.001). BF treatment significantly reduced relative abundance of *Nocardioides* and *Rhodanobacter* by 52.5% (*p* < 0.05) and 57.8% (*p* < 0.05), respectively, and enhanced *Luteimonas* abundance up to 4.5 times that of CF (*p* < 0.001). Compared with BFD treatment, BF reduced the abundance of *Rhodanobacter* and *Luteimonas* by 54.7% (*p* < 0.05) and 24.4% (*p* < 0.05), respectively.

**Figure 3:**
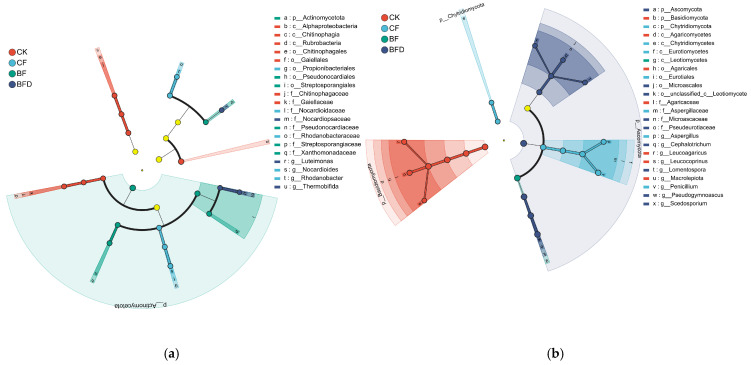
LEfSe analysis (LDA > 4.0, *p* < 0.05) of soil bacterial (**a**) and fungal (**b**) communities under various fertilizer treatments. CK: soil without any fertilizer; CF: chemical fertilizer; BF: bio-organic fertilizer; BFD: deactivated bio-organic fertilizer.

The key microbial taxa for each fertilizer treatment were Identified through LEfSe analysis (LDA > 4, *p* < 0.05). As shown in Figure 3a, the enriched bacterial phyla across all fertilizer treatments primarily belonged to *Actinomycetota*, although the specific subordinate taxa differed among various treatments. The differential taxa in CF treatment were mainly from the order of *Propionibacteriales*, while those in BF treatment were predominantly from the order *Pseudonocardiales* and family *Streptosporangiaceae*. The differential taxa in BFD treatment were chiefly represented by the family *Nocardiopsaceae*.

### 3.4. Effects of Bio-Organic Fertilizer on Fungal Community Composition

The phyla *Basidiomycota*, *Ascomycota*, and *Mucoromycota* were the dominant soil fungal groups, collectively accounting for 94.1–98.5% of the total community. Bio-organic fertilizer exhibited a more pronounced impact on soil fungal community composition than bacterial communities.

As shown in Figure 2c, *Basidiomycota* was the most abundant fungal phylum in the control, whereas *Ascomycota* became the most dominant in fertilizer treatments. The relative abundance of *Ascomycota* in CF, BFD, and BF treatments increased to 1.7 (*p* < 0.05), 1.9 (*p* < 0.01), and 1.9 (*p* < 0.01) times that of CK, respectively. In contrast, relative abundances of *Basidiomycota* in CF, BFD, and BF treatments decreased by 85.7% (*p* < 0.05), 92.2% (*p* < 0.05), and 91.3% (*p* < 0.05), respectively. Furthermore, *Mucoromycota* abundance in BFD and BF treatments decreased by 59.8% (*p* < 0.05) and 50.7% (*p* < 0.05), respectively, compared to the CF treatment.

As illustrated in Figure 2d, *Penicillium* was the most abundant fungal genus in both CK and CF treatments. However, *Pseudogymnoascus* became the dominant one in BFD and BF treatments. The relative abundance of *Penicillium* from BFD and BF treatments decreased by 81.7% (*p* < 0.01) and 82% (*p* < 0.01), respectively, relative to CF treatment. Conversely, relative abundances of *Pseudogymnoascus* in BFD and BF treatments were 7.7 and 6.5 times that of CK, and 3.5 and 3 times that of CF, respectively (*p* < 0.001).

As indicated by Figure 3b, the enriched fungal taxa in each fertilizer treatment significantly differed. The differential taxa in CF treatment primarily belonged to phyla *Ascomycota* (including the genera *Aspergillus* and *Penicillium*) and *Chytridiomycota*. In contrast, BF-treated soil was predominantly enriched with taxa belonging to the class *Leotiomycetes* (within *Ascomycota*). BFD treatment was mainly characterized by the order *Microascales* (also within *Ascomycota*).

### 3.5. Effects of Bio-Organic Fertilizer on Soil Carbon Cycle Functions

The carbon cycle was categorized into carbon degradation, carbon fixation, and methane metabolism (including methanogenesis and oxidation) processes. Among these, carbon fixation and carbon degradation were the primary processes, followed by methane metabolism. Carbon degradation genes primarily include those related to the degradation of starch, cellulose, hemicellulose, lignin, chitin, and pectin.

**Figure 4:**
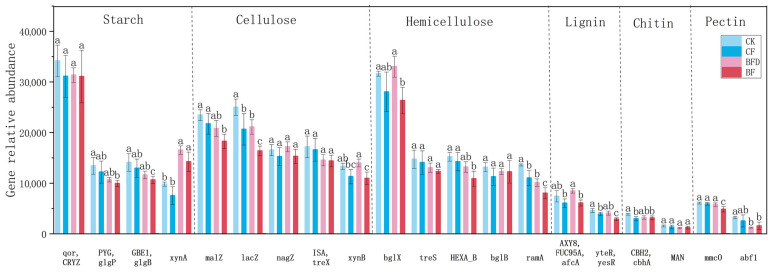
Abundances of key functional genes involved in soil carbon degradation under various fertilizer treatments. CK: soil without any fertilizer; CF: chemical fertilizer; BF: bio-organic fertilizer; BFD: deactivated bio-organic fertilizer. Different letters above columns of degradation of each compound indicate significant differences among treatments.

As shown in Figure 4, for starch degradation-related genes, abundances of *PYG*, *glgP*, *GBE1*, and *glgB* in BF treatment were significantly lower than those of the control. *xynA* abundance was higher in BFD and BF treatments compared to CK and CF treatments. Among cellulose degradation-related genes, *lacZ* abundance in CF treatment was lower than that of CK. Abundances of *malZ*, *lacZ*, and *xynB* in BF treatment were lower than those of the control. *lacZ* abundance in BFD treatment was lower than that of CK, while *xynB* abundance was higher than that of CF treatment. For hemicellulose degradation-related genes, *ramA* abundance in CF and BFD treatments was lower than that of CK. Abundances of *bglX*, *HEXA_B*, and *RamA* in BF treatment were significantly lower than those of CK. Regarding lignin degradation-related genes, abundances of *yteR* and *yesR* in BF treatment were significantly lower than those of the control. For chitin degradation-related genes, abundances of *CBH2* and *cbhA* in BF treatment were lower than those of CK. In terms of pectin degradation genes, the abundances of *mmc0* and *abf1* in BF treatment were significantly lower. Overall, the relative abundance of carbon degradation genes in fertilizer treatments was generally lower than that of the control.

**Figure 5:**
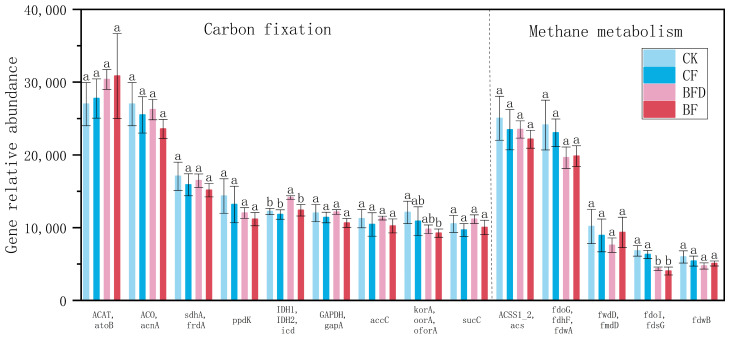
Abundance of key functional genes related to soil carbon fixation and methane metabolism under various fertilizer treatments. CK: soil without any fertilizer; CF: chemical fertilizer; BF: bio-organic fertilizer; BFD: deactivated bio-organic fertilizer. Different letters above columns of each C-cycle process indicate significant differences among treatments.

Carbon fixation genes are primarily involved in the process of converting atmospheric carbon dioxide (CO_2_) into organic carbon compounds. As shown in Figure 5, abundances of *IDH1*, *IDH2*, and *icd* in BFD treatment were significantly higher than those in CK and the other treatments, while those of *korA*, *oorA*, and *oforA* in BF treatment were significantly lower than those in CK. Genes associated with methane metabolism involve two main processes of methanogenesis (methane production) and methane oxidation (methane consumption). The abundances of *fdo1* and *fdsG* in BFD and BF treatments were significantly lower than those in CK and CF treatments, while no significant differences were observed for other genes.

### 3.6. Effects of Bio-Organic Fertilizer on Soil Nitrogen Cycle Functions

The nitrogen cycle mainly includes biological nitrogen fixation, organic nitrogen metabolism, ammonification, nitrification, assimilatory nitrate reduction, dissimilatory nitrate reduction, denitrification, and anaerobic ammonium oxidation.

**Figure 6:**
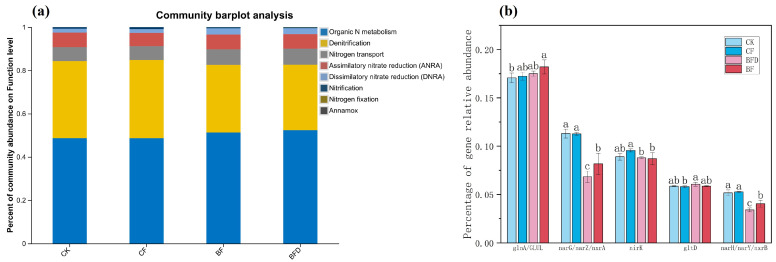
Abundances of overall (**a**) and key (**b**) functional genes of the soil nitrogen cycle under various fertilizer treatments. CK: soil without any fertilizer; CF: chemical fertilizer; BF: bio-organic fertilizer; BFD: deactivated bio-organic fertilizer. Different letters above columns indicate significant differences among treatments.

As shown in Figure 6a, organic nitrogen metabolism and denitrification were the two dominant processes. Compared to CF treatment, the relative abundance of functional genes related to organic nitrogen metabolism increased by 5.3% and 7.6% under BF and BFD treatments, respectively. Conversely, relative abundance associated with denitrification decreased by 13.3% and 16.1% under BF and BFD treatments, respectively. Gene abundance associated with dissimilatory nitrate reduction in BF and BFD treatments was 1.6 times that of CK and 1.5 and 1.6 times that of CF treatment, respectively.

Regarding specific functional genes (Figure 6b), the abundances of ammonia assimilation genes (*glnA*, *GLUL*) in BF treatment were higher than those in CK, while those of nitrate reduction genes (*narG*, *narZ*, *nxrA*) and nitrite oxidation genes (*narH*, *narY*, *nxrB*) were lower than those in CK, with even lower abundances observed in BFD. For the nitrite reductase gene (*nirK*), its abundance in BFD and BF treatments was comparable to CK but lower than that in CF treatment. The abundance of the glutamate synthase gene (*gltD*) in BFD treatment was significantly higher than that in CF treatment.

### 3.7. Correlations Between C/N Cycle Genes and Soil Physicochemical Properties/Enzyme Activities

**Figure 7:**
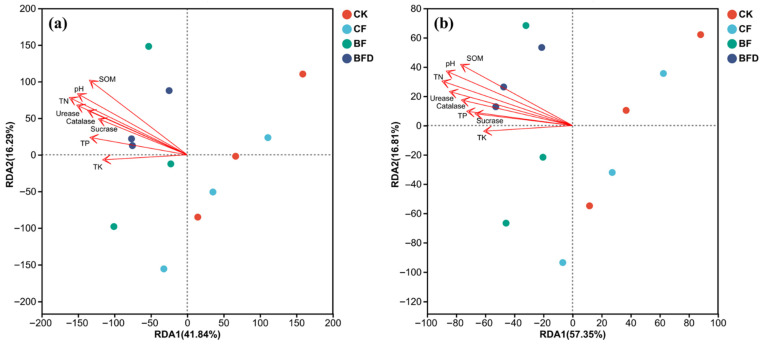
RDA analysis of soil carbon (**a**)/nitrogen (**b**) cycling genes and soil properties/enzyme activities under various fertilizer treatments. CK: soil without any fertilizer; CF: chemical fertilizer; BF: bio-organic fertilizer; BFD: deactivated bio-organic fertilizer.

Soil pH, SOM, TN, as well as urease and catalase activities, significantly influenced soil carbon and nitrogen cycle functions (*p* < 0.05). As shown in Figure 7, bio-organic fertilizer treatments (BF, BFD) were distributed along the positive direction of soil environmental factors and were distinctly separated from CK and CF. This indicates that bio-organic fertilizers drive changes in soil carbon and nitrogen cycle functions by enhancing soil pH, SOM, TN, and enzyme activities.

## 4. Discussion

### 4.1. Effects of Bio-Organic Fertilizer on Soil Properties and Plant Growth

**Figure 8:**
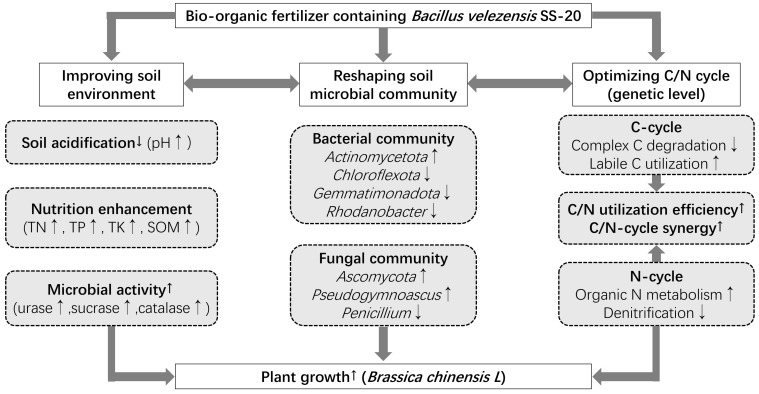
Proposed mechanistic diagram illustrating how bio-organic fertilizer containing *Bacillus velezensis* SS-20 enhances soil health and promotes plant growth. The up-arrow (↑) and down-arrow (↓) indicate significant increase/facilitation and decrease/inhibition respectively.

A mechanism diagram illustrating how bio-organic fertilizer enhances soil health and promotes plant growth is displayed in Figure 8. Bio-organic fertilizer (BF) improved soil physicochemical properties, as indicated by increased pH and significant enhancements in the contents of organic matter, total nitrogen (TN), total phosphorus (TP), and total potassium (TK). For instance, compared to BFD treatment, BF treatment increased soil TN and TP contents by 5.7% and 8.0%, respectively. This improvement may be attributed to the functional strain SS-20 in BF. Our previous study demonstrated that *Bacillus belezensis* SS-20 is capable of dissolving inorganic phosphorus (phosphate solubilization efficiency = 45.95%) and producing siderophore (siderophore units = 54.18 ± 4.19%) and poly-*γ*-glutamic acid (13.35 ± 2.61 g/L) [24]. This strain was also found to increase plant height, aboveground fresh weight, and root dehydrogenase activity of Chinese cabbage. As key indicators of soil metabolic activity, changes in soil enzyme activities effectively reflect microbial vitality. Soil enzyme activities, such as urease, sucrase, and catalase, were significantly enhanced under BF treatment. Among these, urease activity showed the most pronounced enhancement, reaching 2.9 times that of CK. Soil urease acts as a “gateway enzyme” in the nitrogen cycle, and its elevated activity indicates the activation of soil nitrogen transformation [25]. Urease catalyzes the hydrolysis of urea to generate ammonia, providing a direct nitrogen source for plants. The ammonium ions generated also contribute to stabilizing soil pH [26]. The synergistic enhancement of sucrase and catalase activities collectively reflects the active metabolic state of soil microorganisms under BF treatment, fostering a microenvironment conducive to organic matter decomposition and nutrient release.

The yield of pakchoi treated with BF (28,047.10 kg/ha) was 11.1% and 10% higher than that of CF and BFD treatments. Pakchoi yield was significantly positively correlated with soil organic matter, total elements (N, P, K), and urease activity. Therefore, bio-organic fertilizers can enhance the metabolic activity of microorganisms and accelerate nutrient release, thereby promoting plant growth. In addition, the strain SS-20 in bio-organic fertilizer can synthesize and secrete polyglutamic acid (which can improve fertilizer utilization) and auxin (which can regulate the growth and development of plant organs), which further improves nutrient utilization and plant growth [24]. The differential effects among treatments underscore the synergy between organic matter and functional microbes. While both BF and BFD (containing organic matter) significantly improved soil pH and nutrient content over CF, BF treatment led to higher TN, TP, and enzyme activities than BFD. This indicates that organic matter input alone can mitigate acidification and enhance nutrient retention, but the active SS-20 strain in BF is crucial for maximizing nutrient cycling efficiency and plant growth promotion. Although BFD treatment was superior to chemical fertilizer (CF) in some soil properties, it did not significantly improve pakchoi yield compared with CF, indicating a key role of active microorganisms in promoting plant growth.

### 4.2. Soil Microbial Community Structure Shifted by Bio-Organic Fertilizer

Compared to CK and CF treatments, soil microbial communities in both BF and BFD treatments were significantly distinct, indicating a reshaping effect of bio-organic fertilizer on soil microbial communities. However, the extent and functional direction of this reshaping differed. BF induced a more pronounced and beneficial shift compared to BFD. At the bacterial phylum level, the relative abundance of *Actinomycetota* increased in BF treatment compared to BFD. This phylum plays a significant role in the degradation of recalcitrant organic matter [27]. Its increased abundance may correlate with the observed enhancement in soil enzyme activity, thereby promoting nutrient release and plant growth. Meanwhile, abundances of *Chloroflexota* and *Gemmatimonadota* were reduced in both BF and BFD treatments, and the reduction in BF was more significant. These phyla are associated with oligotrophic environments [28], so their decline may reflect a shift in the microbial community from an oligotrophic to a copiotrophic state. Compared to CF treatment, BFD treatment reduced abundances of *Acidobacteriota* and *Gemmatimonadota*. The preference of *Acidobacteriota* for acidic environments aligns with its decrease following the observed rise in soil pH (Table 2). At the bacterial genus level, the relative abundance of *Luteimonas* significantly increased in both BF and BFD treatments, suggesting its sensitive response to organic matter input [29]. Furthermore, some strains within this genus have been reported to have the potential for phosphate solubilization and production of plant growth hormones such as IAA [30], which could be the mechanism of plant growth promotion. Notably, the abundance of *Luteimonas* was lower in BF treatment than that in BFD, indicating that the increased pakchoi yield does not rely solely on a single genus but on the functional balance and synergistic interactions of the entire microbial community. Overall, bio-organic fertilizer optimized the microbial community structure by enriching key functional phyla (e.g., *Actinomycetota*) and regulating other nutrient status-related microbial groups, thereby promoting soil nutrient cycling and plant growth [31].

In this study, *Bacillus* did not belong to the top-ten dominant taxon in BF treatment, but their role in regulating the microbial community should not be ignored. Some functional microbes, even at low abundances, can significantly influence overall community function by regulating the community structure and activating specific metabolic pathways, or secreting key signaling molecules and acting as “keystone species” or “starter factors” [32,33]. For instance, Tao et al. [34] found that bio-organic fertilizer containing *Bacillus amyloliquefaciens* W19 significantly enhanced plant disease suppression, although *Bacillus* is not the dominant top-ten taxa in soil. The effect mainly results from functional activation (such as inducing beneficial bacteria such as *Pseudomonas*) rather than quantitative dominance. Future research could employ techniques (such as GFP labeling, in situ fluorescence imaging, and functional gene tracking) to further elucidate the activity and mechanistic roles of these low-abundance functional microbes in soil, thereby providing a more comprehensive understanding of the intrinsic mechanisms by which bio-organic fertilizers regulate soil microecology.

The fungal community responded more pronouncedly than bacteria to BF and BFD treatments. The relative abundance of *Basidiomycota* decreased significantly, while that of *Ascomycota* increased markedly in both BF and BFD treatments. Since *Ascomycota* encompasses numerous saprophytic fungi capable of decomposing cellulose and lignin [28], its substantial increase is likely related to the organic fertilizer input. This enhances soil enzyme activities, such as sucrase, and promotes nutrient release. Concurrently, the abundance of *Mucoromycota* was lower in BF and BFD treatments compared to CF. Given that this phylum may include certain pathogenic fungi (e.g., *Rhizopus oryzae*, *Mucor* spp.) [35,36], its reduction may have indirectly contributed to the yield increase of pakchoi. *Pseudogymnoascus* became the dominant genus in both BF and BFD treatments. This genus possesses capabilities for antagonizing pathogens or decomposing organic matter [37,38], and its increase is likely associated with enhanced soil biocontrol potential and nutrient cycling. In contrast, the abundance of *Penicillium* decreased in BF and BFD treatments compared to CF. As this genus includes pathogenic or mycotoxin-producing strains [39,40], its reduction may have lowered plant disease pressure and led to increased yield. Our previous research has found that SS-20 can secrete secondary metabolites, such as surfactin, which confer antagonistic activity against several fungal pathogens. Specifically, SS-20 can inhibit common fungal diseases such as rice blast, sheath blight, and fusarium wilt [24]. Overall, BF treatment shifted the fungal community toward a more beneficial composition. The increase in *Ascomycota* and *Pseudogymnoascus* enhanced organic matter decomposition and biocontrol potential, while the decrease in *Penicillium* reduced disease risk. These alterations directly supported the observed improvements in soil enzyme activities and nutrient contents.

LEfSe analysis indicated that the differential microbial taxa in BF treatment primarily included bacteria from the order *Pseudonocardiales* and family *Streptosporangiaceae*, as well as fungi from class *Leotiomycetes* and order *Diaporthales*. The order *Pseudonocardiales* is widely involved in organic matter decomposition and nutrient transformation [41]. The family *Streptosporangiaceae* is known for its strong decomposing ability of complex organic materials such as cellulose and chitin [42,43]. The order *Diaporthales* produces enzymes that degrade plant residues, which may accelerate nutrient release [44]. The synergistic effects of these microbial communities contributed to the significant advantage of BF treatment in increasing pakchoi yield. Nevertheless, the hypothesis that SS-20 recruits functional microorganisms and that the microbial community shift drives functional improvements still requires further research.

### 4.3. Regulation Mechanism of Bio-Organic Fertilizer on Soil Carbon Cycle

The abundance of soil carbon degradation genes generally decreased following fertilizer application. In BF treatment, the abundances of most carbon degradation genes were significantly lower than those in the control, including those involved in the degradation of starch (e.g., *PYG*, *glgP*, *GBE1*, *glgB*), cellulose (e.g., *malZ*, *lacZ*, *xynB*), hemicellulose (e.g., *bglX*, *HEXA_B*, *RamA*), lignin (e.g., *yteR*, *yesR*), chitin (e.g., *CBH2*, *cbhA*), and pectin (e.g., *mmc0*, *abf1*). The reduction is likely due to the readily degradable organic carbon from BF treatment, allowing microorganisms to directly utilize these carbon sources instead of complex materials. Consequently, microbial communities may have shifted towards a more efficient metabolic strategy, reducing gene abundance encoding enzymes to degrade complex carbon sources. This aligns with the observed increase in soil enzyme activity, which stems from the rapid utilization of readily degradable carbon rather than the degradation of complex carbon compounds. This shift was most evident in BF. BFD treatment also showed a reduction in some carbon degradation genes, but to a lesser extent, and even an increase in certain genes like *xynA*. This contrast suggests that the presence of active SS-20 in BF more effectively redirects microbial metabolism toward the use of readily available carbon, whereas in BFD, the microbial community may still partially rely on decomposing more complex substrates from the added organic matter.

Changes in carbon fixation genes were not significant overall. Abundances of *korA*, *oorA*, and *oforA* genes in BF treatment were significantly lower than those in CK, whereas BFD treatment showed higher abundances of *IDH1*, *IDH2*, and *icd* genes. BF treatment provided exogenous organic carbon and potentially reduced microbial dependence on CO_2_ fixation, thus leading to a lower abundance of carbon fixation genes. However, the higher abundance of carbon fixation genes in BFD treatment may be attributed to the reduced microbial competition after deactivating treatment [45], thereby stimulating carbon fixation activities in certain autotrophic microorganisms.

The abundances of *fdo1* and *fdsG* involved in methane oxidation were significantly lower in BF and BFD treatments compared to CK and CF treatments, indicating suppression of the methane oxidation process. This may be attributed to changes in soil redox potential. The increased pH and nutrient elements under BF treatment likely improved soil aeration and reduced anaerobic conditions, thereby inhibiting methanogen activity. Simultaneously, methanotroph activity may have decreased due to reduced methane substrate.

Overall, BF impacted the carbon cycle by improving soil properties (pH, nutrient levels), enhancing enzyme activities, and modulating microbial community functions. BF treatment likely promoted a “fast carbon cycle,” in which microorganisms directly utilized readily degradable organic carbon, accelerating organic matter transformation and nutrient release. Its effects were superior to those of CF and BFD, underscoring the critical role of functional microorganisms in driving the carbon cycle.

### 4.4. Regulation Mechanism of Bio-Organic Fertilizer on Soil Nitrogen Cycle

Gene abundances associated with soil organic nitrogen metabolism were increased under BF treatment. Organic nitrogen metabolism primarily refers to the process by which microorganisms break down macromolecular organic nitrogen compounds (such as proteins and nucleic acids) into ammonium nitrogen. BF supplies a substantial amount of organic matter, which serves as an adequate food source for ammonifying microorganisms, thereby stimulating the proliferation of related functional genes and microbial populations. Furthermore, the ammonification process consumes H^+^, which aligns with the observation that BF treatment significantly elevates soil pH. In contrast, the abundance of denitrification functional genes was reduced in BF treatment. Denitrification is the anaerobic reduction of nitrate (NO_3_^-^) to gaseous nitrogen forms (N_2_, N_2_O), representing a major pathway for nitrogen loss from farmland and a source of greenhouse gas emissions. The decreased abundance of related genes indicates a reduced risk of nitrogen loss via gaseous forms, thereby retaining more nitrogen in soil for plant uptake. This contributes to enhanced soil nitrogen content and increased pakchoi yield under BF treatment.

Changes in the microbial community structure drove functional shifts in the nitrogen cycle. Compared with BFD, BF treatment significantly increased *Actinomycetota* abundance. *Actinomycetota* are typical decomposers of organic matter, especially proficient in degrading recalcitrant organic compounds (e.g., cellulose and chitin), and likely serve as key drivers of organic nitrogen metabolism. *Luteimonas* was significantly enriched in both BF and BFD treatments; this genus is associated with phosphate solubilization and plant growth promotion and may also participate in ammonification. Certain species from *Rhodanobacter*, known to be involved in denitrification [46,47], showed significantly lower abundance in BF compared to BFD, aligning with the observed decrease in the abundance of denitrification functional genes. For the fungal community, BF treatment established *Ascomycota* as the predominant phylum. *Ascomycota* encompasses a wide range of saprophytic fungi capable of decomposing recalcitrant organic materials such as lignin. Together with *Actinomycetota*, they promoted the mineralization of organic nitrogen under BF treatment. The genus *Pseudogymnoascus* became dominant in BF treatment. Typically found in organic-rich soils and possessing strong decomposing ability, its predominance over *Penicillium* likely contributed to improved soil health and pakchoi growth. This suggests that SS-20 in BF not only directly contributes to nitrogen metabolism but also modulates the wider community, enriching taxa like *Actinomycetota* that support nitrogen mineralization while suppressing denitrifiers like certain *Rhodanobacter* species more effectively than BFD does.

The enhanced activities of catalase, urease, and sucrase observed in BF treatment may be related to shifts in the microbial community structure and the nitrogen cycle. Urease directly participates in the decomposition of organic nitrogen compounds such as urea, and its increased activity is a direct indicator of vigorous organic nitrogen metabolism [48]. Sucrase reflects the intensity of the soil carbon cycle. The organic matter supplied by BF stimulated microbial activity, and the enhanced carbon cycle provided an energy source for microorganisms involved in the nitrogen cycle. Catalase is linked to aerobic microbial metabolism [49,50]. Its elevated activity suggests that the overall microbial metabolism was more active under BF treatment, which facilitated the decomposition and transformation of organic matter.

### 4.5. Correlation Analysis of Soil C/N Cycle Under Bio-Organic Fertilizer Treatment

Compared with the control, the abundance of most carbon-degrading genes was reduced under BF treatment. This reduction spanned nearly all pathways for degrading complex organic matter, including starch, cellulose, hemicellulose, lignin, chitin, and pectin. Degrading complex organic carbon is an energy-intensive process [51]. The microbial nitrogen cycle (such as organic nitrogen metabolism and ammonia assimilation) requires substantial energy and carbon skeletons. Under BF treatment, microorganisms no longer need to invest significant effort in synthesizing complex enzymes to utilize native recalcitrant organic carbon in soil. Instead, they directly utilized the readily degradable carbon sources from organic fertilizer to supply energy for the nitrogen cycle, thereby synergistically enhancing the efficiency of both carbon and nitrogen turnover.

However, an exception was observed for *xynA*, a gene involved in degrading hemicellulose, which is one of the relatively more degradable components of plant cell walls. Its abundance was higher in both BF and BFD treatments compared to CK and CF. This exception indicates that microorganisms selectively targeted certain readily degradable components. The retention of the *xynA* gene may enable microbes to rapidly utilize hemicellulose-containing organic fragments, either from organic fertilizer or crop root exudates, to quickly acquire carbon and energy. This rapid carbon acquisition, in turn, could drive accelerated nitrogen transformations.

Carbon fixation and nitrogen assimilation are two fundamental processes for microbial growth. The upregulation of certain carbon fixation genes under BFD treatment may represent a “stress response”. The deactivated organic fertilizer provides nutrients but lacks functional microorganisms, potentially stimulating the native microbes to enhance carbon fixation to meet growth demands. In contrast, BF supplies abundant, readily available carbon and nitrogen sources along with live microorganisms. Microbes can directly utilize these substrates for growth, thus exhibiting a relatively lower demand for carbon fixation. This reflects a better match between carbon/nitrogen inputs and microbial requirements in BF-treated soil. The increased abundance of ammonia assimilation genes (*glnA*, *GLUL*) in BF treatment is a clear indication that functional microorganisms, including SS-20, are actively assimilating inorganic nitrogen into their own biomass. This process is tightly coupled with the utilization of exogenous organic carbon for biomass synthesis [24].

Overall, the changes in carbon and nitrogen cycle genes under BF treatment reveal a microbial co-metabolism strategy. By using exogenous, easily degradable organic carbon as the primary substrate, this strategy drives nitrogen mineralization and assimilation while simultaneously minimizing inefficient losses of carbon and nitrogen nutrients. This efficient strategy constitutes the mechanism through which BF treatment significantly enhances soil fertility and plant yield. Compared to BFD (the deactivated treatment), the live microorganisms in BF play a key role in better regulating this coupled process.

## 5. Conclusions

This study demonstrates that bio-organic fertilizer (BF) containing the functional strain *Bacillus velezensis* SS-20 significantly enhances soil fertility and promotes plant growth through integrated improvements in soil properties, the microbial community structure, and carbon–nitrogen cycle functions. Key mechanisms include mitigating soil acidification, increasing key enzyme activities, and enriching beneficial microbes such as *Actinomycetota* and *Pseudogymnoascus*, while suppressing harmful genera. At the functional gene level, BF promotes the coupling of C and N cycles by enhancing the use of readily available organic carbon and boosting organic nitrogen metabolism while inhibiting denitrification, thereby improving nutrient retention and availability. These integrated effects collectively lead to a significant increase in pakchoi yield, highlighting the superiority of BF over conventional chemical fertilizer and deactivated organic fertilizer, and underscoring the essential role of live functional microorganisms in driving sustainable soil fertility and crop productivity. This study provides mechanistic support for the application of BF as a sustainable agricultural practice. Future research needs to focus on elucidating the interactions between the introduced strain and native microorganisms and evaluating long-term effects and economic feasibility under diverse conditions. Multi-omics approaches can be utilized to unravel microbial network dynamics and develop tailored multi-strain formulations for enhanced resilience and efficiency.

## Figures and Tables

**Table 1:** Physicochemical properties of the original soil.

Soil Property	pH	OM (g/kg)	TN (g/kg)	TP (g/kg)	TK (g/kg)
Original soil	6.16	11.19	1.31	1.14	1.52

**Table 2:** Soil physicochemical properties and yields of Chinese cabbage under various fertilizer treatments.

Treatment	pH	OM (g/kg)	TN (g/kg)	TK (g/kg)	TP (g/kg)	Yields of Chinese Cabbage (kg/ha)
CK	4.99 ± 0.05 b	18.93 ± 1.60 c	1.37 ± 0.04 c	9.86 ± 1.73 b	0.77 ± 0.10 c	19,339.15 c
CF	4.76 ± 0.01 c	16.92 ± 0.92 c	1.42 ± 0.02 c	11.75 ± 2.28 ab	0.93 ± 0.22 bc	25,233.75 b
BF	5.73 ± 0.02 a	29.62 ± 1.26 a	2.03 ± 0.09 a	13.64 ± 0.41 a	1.22 ± 0.59 a	28,047.10 a
BFD	5.85 ± 0.12 a	26.40 ± 1.26 b	1.92 ± 0.04 b	12.56 ± 1.58 ab	1.13 ± 0.13 ab	25,497.05 b

Different letters in the same column indicate significant differences between treatments. CK: soil without any fertilizer; CF: chemical fertilizer; BF: bio-organic fertilizer; BFD: deactivated bio-organic fertilizer.

**Table 3:** Soil catalase, urease, and sucrase activities under various fertilizer treatments.

Treatments	Catalase Activity (U·g^−1^)	Urease Activity (μg NH_3_-N·g^−1^·h^−1^)	Sucrase Activity (mg glucose·g^−1^·24 h^−1^)
CK	33.36 ± 2.11 c	240.64 ± 13.85 c	32.69 ± 1.89 c
CF	27.95 ± 2.97 d	195.78 ± 22.10 d	21.10 ± 1.40 d
BF	48.79 ± 1.14 a	684.55 ± 14.12 a	50.76 ± 0.91 a
BFD	41.37 ± 1.51 b	538.73 ± 14.27 b	35.88 ± 1.84 b

Different letters in the same column indicate significant differences among treatments. CK: soil without any fertilizer; CF: chemical fertilizer; BF: bio-organic fertilizer; BFD: deactivated bio-organic fertilizer.

## Data Availability

The original contributions presented in this study are included in the article/Supplementary Materials. Further inquiries can be directed to the corresponding authors.

## Supplementary Materials

The following supporting information can be downloaded at: https://www.mdpi.com/article/10.3390/plants15030382/s1, Table S1: Correlation analysis of soil physicochemical property/enzyme activity and yields of Chinese cabbage; Table S2: *α* diversity of soil bacteria and fungi under different fertilizer treatments.
