# Supplementary material for: Bio-Organic Fertilizer with Bacillus velezensis Promoted Plant Growth by Regulating Soil Microbial Community Structure and C/N Cycle Function
Source: Plants (Basel). 2026 Jan 26;15(3):382. doi: 10.3390/plants15030382 (PMC12899839; doi:10.3390/plants15030382)
Supplement: Supplementary file 1 [file plants-15-00382-s001.zip › plants-4069217-supplementary.pdf]

Bio-Organic Fertilizer with *Bacillus velezensis* Promoted Plant Growth by Regulating Soil Microbial Community Structure and C/N Cycle Function

Haiyun Zhang [1,2,3], Cuixue Cui [1,2,3,4], Shuangxi Li [1,2,3], Weiguang Lv [1,2,3], Juanqin Zhang [1,2,3], Xianpu Zhu [1,2,3], Chenglong Xu [1,2,3], Qun Wang [5], Naling Bai [1,2,3,*] and Hanlin Zhang [1,2,3,*]

1 Institute of Eco-Environmental Protection, Shanghai Academy of Agricultural Sciences, Shanghai 201403, China; zhanghaiyun@saas.sh.cn (H.Z.)
2 Shanghai Agri-Environmental and Cultivated Land Conservation of Scientific Observation and Experiment Station, Ministry of Agriculture and Rural Affairs, Shanghai 201403, China
3 Key Laboratory of Low-Carbon Green Agriculture in Southeastern China, Ministry of Agriculture and Rural Affairs, Shanghai 201403, China
4 School of Chemical and Environmental Engineering, Shanghai Institute of Technology, Shanghai 201418, China
5 Key Laboratory of Recycling and Eco-Treatment of Waste Biomass of Zhejiang Province, School of Environmental and Natural Resources, Zhejiang University of Science and Technology, Hangzhou 310023, China
* Correspondence: bainaling@saas.sh.cn (N.B.); zhanghanlin@saas.sh.cn (H.Z.); Tel.: +86-21-62202484 (N.B.); +86-21-62202607 (H.Z.)

Supporting Information

Number of tables: 2

Table S1 Correlation analysis of soil physicochemical property/enzyme activity and yields of Chinese cabbage.

		Yields of Chinese cabbage (kg/ha)	OM (g/kg)	TN (g/kg)	TK (g/kg)	TP (g/kg)	Urease activity (μg NH_3-N·g^{-1}·h^{-1})	Catalase activity (U·g^{-1})	Sucrase activity (mg glucose·g^{-1}·24 h^{-1})
Yields of Chinese cabbage (kg/ha)	*r*	1	0.611*	0.761**	0.715**	0.752**	0.685*	0.528	0.417
	p		0.035	0.004	0.009	0.005	0.014	0.078	0.177
	n	12	12	12	12	12	12	12	12

Table S2 α diversity of soil bacteria and fungi under different fertilizer treatments.

Treatments	Bacteria			Fungi		
	Chao	Shannon	Pielou_e	Chao	Shannon	Pielou_e
CK	34249±192a	7.404±0.046a	0.625±0.069a	1351±110b	4.498±0.447a	0.709±0.004a
CF	33792±327a	7.316±0.088a	0.661±0.039a	1307±74b	4.743±0.248a	0.702±0.008a
BF	33878±530a	7.354±0.241a	0.704±0.019a	1446±80ab	4.962±0.424a	0.705±0.022a
BFD	34227±226a	7.492±0.043a	0.682±0.063a	1526±17a	5.160±0.138a	0.718±0.004a

Different letters in the same column indicate significant differences between treatments.
